# Urochordate Ascidians Possess a Single Isoform of Aurora Kinase That Localizes to the Midbody via TPX2 in Eggs and Cleavage Stage Embryos

**DOI:** 10.1371/journal.pone.0045431

**Published:** 2012-09-20

**Authors:** Celine Hebras, Alex McDougall

**Affiliations:** Université Pierre et Marie Curie and CNRS, Developmental Biology Unit, Villefranche-sur-Mer, France; Institut de Génétique et Développement de Rennes, France

## Abstract

Aurora kinases are key proteins found throughout the eukaryotes that control mitotic progression. Vertebrate Aurora-A and B kinases are thought to have evolved from a single Aurora-kinase isoform closest to that found in present day urochordates. In urochordate ascidians Aurora binds both TPX2 (a vertebrate AURKA partner) and INCENP (a vertebrate AURKB partner) and localizes to centrosomes and spindle microtubules as well as chromosomes and midbody during both meiosis and mitosis. Ascidian Aurora also displays this localization pattern during mitosis in echinoderms, strengthening the idea that non-vertebrate deuterostomes such as the urochordates and echinoderms possess a single form of Aurora kinase that has properties of vertebrate Aurora-kinase A and B. In the ascidian, TPX2 localizes to the centrosome and the spindle poles also as in vertebrates. However, we were surprised to find that TPX2 also localized strongly to the midbody in ascidian eggs and embryos. We thus examined more closely Aurora localization to the midbody by creating two separate point mutations of ascidian Aurora predicted to perturb binding to TPX2. Both forms of mutated Aurora behaved as predicted: neither localized to spindle poles where TPX2 is enriched. Interestingly, neither form of mutated Aurora localized to the midbody where TPX2 is also enriched, suggesting that ascidian Aurora midbody localization required TPX2 binding in ascidians. Functional analysis revealed that inhibition of Aurora kinase with a pharmacological inhibitor or with a dominant negative kinase dead form of Aurora caused cytokinesis failure and perturbed midbody formation during polar body extrusion. Our data support the view that vertebrate Aurora-A and B kinases evolved from a single non-vertebrate deuterostome ancestor. Moreover, since TPX2 localizes to the midbody in ascidian eggs and cleavage stage embryos it may be worthwhile re-assessing whether Aurora A kinase or TPX2 localize to the midbody in eggs and cleavage stage embryos.

## Introduction

Cell division is controlled by key protein kinases including cyclin-dependent kinase 1 (Cdk1), polo like kinases (Plk) and Aurora kinases. These kinases control the orderly processes of nuclear envelope breakdown, chromosome condensation, mitotic spindle formation, kinetochore-microtubule attachment and cytokinesis (reviewed by [1]). Aurora kinase is a family of serine/threonine protein kinases that is found throughout the eukaryotes [2]. Two major forms of Aurora kinase exist (Aurora-A and B kinase) that exhibit distinct subcellular localizations consistent with their roles [3]. In vertebrates Aurora-A kinase localizes to spindle microtubules through association with TPX2 (Targeting protein for *Xenopus* kinesin-like protein 2) [4]. Aurora-B kinase binds INCENP and is part of the chromosomal passenger complex (CPC) which moves from the inner kinetochore to the central spindle and midbody at anaphase [5]. Starfish possess a single isoform of Aurora that localizes to centrosomes, spindles and chromosomes indicating that it behaves as Aurora-A and B kinase [6]. Although it has yet to be tested, it is possible that the ancestor of vertebrate Aurora-A and B kinase could bind to either TPX2 or INCENP, thereby explaining how starfish Aurora-kinase displays both an Aurora-A and B kinase localization pattern.

Studying the evolution of Aurora within the deuterostomes has suggested that vertebrate Aurora-A and B kinases evolved from a common ancestor shared with non-vertebrate deuterostomes [7]. Amongst the invertebrates, urochordates are the closest living relatives of vertebrates [8] and molecular phylogenetic analysis has placed urochordate Aurora closest to vertebrate Auroras [7]. However, all eukaryotes studied possess Aurora kinase. The yeasts *S. cerevisiae* and *S. pombe* each have one isoform of Aurora: Ipl1 and Ark1 respectively [9]; [10]. The slime mould *Dictyostelium* has a unique isoform of Aurora that has properties of both Aurora-A and B kinase and has therefore been suggested to be closer to the ancestral form of Aurora [11]. In metazoans Aurora-A kinase (DmAurora) was initially described in *Drosophila* [12]. A second isoform was later discovered in *Drosophila* (IAL) that has properties similar to Aurora-B kinase [13]. Like *Drosophila, C. elegans* has two isoforms of Aurora termed AIR-1 and AIR-2 that are grouped as Aurora-A and B kinases [14], [15]. Vertebrates also have Aurora-A and B kinase while mammals possess a third isoform termed Aurora-C kinase that is closely related to Aurora-B kinase [16]. However, although protostomes (*Drosophila* and *C. elegans*) have two isoforms of Aurora (designated types A and B), it came as a surprise to find that non-vertebrate deuterostomes such as echinoderms [6], hemichordates, cephalochordates and the urochordates represented by the ascidian have only one isoform of Aurora. The unique isoform of Aurora found in these non-vertebrate deuterostomes may therefore be similar to the ancestor of vertebrate Aurora-A and B kinases [7]. This is supported by the finding that the unique isoform of Aurora kinase in starfish behaves as both Aurora-A and B kinase [6]. The corollary of this is that the two protostome Aurora isoforms (A and B in *Drosophila* and *C. elegans*) likely arose by a separate gene duplication event after the protostomes and deuterostomes diverged [7]. Non-vertebrate deuterostomes therefore represent an appropriate model system to determine how the last common ancestor of invertebrate and vertebrate Aurora kinases may have behaved. And equally important, understanding how the ancestor of vertebrate Aurora-A and B kinases behaved may help us better understand some aspects of the two vertebrate proteins.

One of the functions of Aurora is to regulate cytokinesis, a multistep process that culminates in the physical separation of two daughter cells by abscission (reviewed by [17]). Failure to physically separate daughter cells results in tetraploidy and promotes tumorigenesis [18]. An abscission checkpoint mediated by Aurora-B kinase delays abscission if chromosome bridges are present thereby reducing the risk of tetraploidy [19]. Unfortunately Aurora-B kinase is involved in other mitotic processes making it very difficult (in animal cells) to perform knockdown, knockout, or even dominant negative experiments to explore the late mitotic functions of Aurora-B kinase. To identify the role played by Aurora-B kinase at the midbody in higher vertebrates one approach has been to apply fast-acting pharmacological inhibitors of Aurora-B kinase such as hesperidin or ZM447439 following spindle formation and chromosome segregation [19].

Oocytes are an attractive model to study abscission which also occurs during polar body cytokinesis [20]. One of the reasons for this is that oocytes are often blocked in metaphase I or II awaiting fertilization with fully formed spindles making it possible to perturb Aurora function in oocytes that have already assembled their spindles. Another advantage of oocytes for the study of abscission is that very little furrowing (which also involves Aurora-B kinase) is required to bring the plasma membranes close to the midbody since the spindle is already located subjacent to the cortex. Finally, some oocytes such as the ascidian do not display a spindle attachment checkpoint [21], making it possible to inhibit Aurora function without perturbing mitotic progression. In mouse oocytes Aurora-A is the most abundant form or the three Aurora-kinases A, B and C [22] and is stable during meiosis I when the first polar body is being extruded [23]. In bovine oocytes, Aurora-A kinase is localized to the midbody during first polar body extrusion [24]. In mouse oocytes Aurora-C kinase localizes to meiotic chromosomes and the midbody and knockdown of Aurora-C kinase causes premature separation of homologous chromosomes, an increase in the frequency of merotelic and syntelic chromosomal configurations, the entrapment of chromosomes in the neck of the polar body at anaphase I and failure to cleave the extruded polar body which subsequently re-absorbs [25]. It is not yet known whether this cytokinesis defect is due to chromosomal bridges which may impede abscission as in somatic cells [19], or whether some other midbody function is affected.

Here we have investigated urochordate ascidian Aurora which is thought to be the closest form of non-vertebrate deuterostome Aurora to vertebrate Aurora-A and B kinases. We show that ascidian Aurora has a localization pattern similar to vertebrate Aurora-A and B kinases, and that ascidian Aurora binds both INCENP (an Aurora-B kinase interacting protein) and TPX2 (an Aurora-A kinase interacting protein). To our surprise we also found that TPX2 localizes to the midbody in urochordate ascidian embryos (in vertebrates most reports show that TPX2 does not localize to the midbody), suggesting that vertebrate Aurora A and B differential subcellular localization pattern is in part brought about because vertebrate TPX2 lost the ability to localize to the midbody (although this does not explain Aurora A localization to the midbody in bovine oocytes). Finally, we exploited oocyte cytokinesis (polar body formation) to study the role of Aurora during midbody formation. We show that inhibiting ascidian Aurora function blocks cytokinesis in oocytes (as it does in embryos) likely due to failure to form a midbody.

## Materials and Methods

### Biological Material

Eggs from the ascidian *Phallusia mammillata* were harvested from animals obtained in Sète and kept in the laboratory in a tank of natural sea water at 16°C. Egg preparation and microinjection have been described previously (see detailed protocol in [26]). All imaging experiments were performed at 19°C.

### Molecular Methods and Tools

All constructs were made using pSPE3 [27] and the Gateway cloning system (Invitrogen) unless otherwise stated (see [26] for a detailed protocol). Ci-Aurora (KH.C7.219.v1.A.SL1-1), Ci-INCENP (the gene model KH.C14.611.v1.B.SL1-1 was found to be truncated so we cloned full length Ci-INCENP by using the Gilchrest dataset), Ci-TPX2 (KH.C3.181.v1.A.SL1-1), and Ci-Plk1 (KH.C12.238.v1.A.SL1-1) were all sub-cloned into pSPE3 to create either N or C terminal Venus/mRfp1/mCherry fusion constructs. For all fluorescent fusion protein constructs we indicate the N or C terminal position of the fluorescent protein (FP) by the following convention: FP::protein X or protein X::FP.

Imaging of microtubules (to visualize spindles) was achieved by expression of microinjected mRNA coding for the microtubule binding domain of Map7 protein fused to Venus fluorescent protein while histone H2B::Rfp1 was used to follow DNA [28].

Synthetic mRNAs were prepared as concentrated solutions (>2 µg/µl) in distilled water and small aliquots were frozen at −80°C. Solutions were centrifuged for 15 minutes at 13,000 rpm in a microfuge prior to loading the needle in order to sediment particles that might block the injection needle. Fluorescence from fusion protein constructs was observed in an unfertilized egg approx. 5 hours after injection of concentrated mRNAs and a few hour after injection into a zygote.

Site-directed mutagenesis of Ci-Aurora was performed to create Ci-Aurora S60R, Ci-Aurora G103N and a dominant negative form of Ci-Aurora (T193A/T247D/S248D, based on [3] using the Quickchange kit (Stratagene) following the manufacturers protocol. After site-directed mutagenesis the mutants were cloned using the Gateway system.

GST::Aurora fusion protein was made using a derivative of the pGEX expression vector system (pGEX4T1). Following bacterial expression, the protein was purified by GST-affinity chromatography according to the kit protocol (Qiagen). GST-Aurora was retained on the Sephadex beads for later interaction with radioactive ^35^S-labeled proteins. To prepare ^35^S-labeled proteins the rabbit reticulocyte lysate system was used according to the manufacturers protocol (Promega). Briefly, the reticulocyte lysate system (Promega) was supplemented with ^35^S and mRNA encoding Ci-TPX2 or Ci-INCENP and the proteins made according to the manufacturer’s instructions. Interaction between Ci-GST::Aurora and target proteins was confirmed by migration of the interacting proteins on 12% polyacrylamide gels followed by autoradiography.

### Use of Pharmacological Inhibitors and Immunofluorescence

Treatments with 10 µM ZM447439 (Tocris) was carried out by bathing unfertilized eggs in sea water supplemented with the drug for 30 min. ZM447439 inhibits both Aurora A and B [29]. For immunofluorescence oocytes or embryos were fixed in methanol/1%formaldehyde (−20°C) overnight. They were then progressively rehydrated in PBS/0.02%Triton, permeabilized in PBS/0.25%Triton for 15 min. at RT then incubated in primary and secondary antibodies in PBS/1%BSA overnight at 4°C, with four 15 min. washes with PBS/0.1%Tween following each incubation. Primary antibodies were used at a dilution of 1∶200 unless otherwise indicated and were as follows: anti <$>\scale 60%\raster="rg1"<$>-tubulin (DM1A; Sigma-Aldrich 1∶1000); anti phosphorylated Aurora (ab183318, Abcam), anti-TPX2 (ab70237, Abcam) and anti- INCENP (ab12183, Abcam). Mouse TRITC secondary antibody (Jackson), and rabbit FITC secondary antibody (Jackson) were diluted 1∶50. To stain DNA, following immunofluorescence labelled oocytes/embryos were mounted in VectaShield that contains DAPI (Vector Laboratories, H1200).

### Microinjection and Imaging

Microinjection was performed as previously described [30]. Briefly, dechorionated oocytes were mounted in glass wedges and injected with mRNA (1–2 µg/µl pipette concentration/2–5% injection volume) using a high pressure system (Narishige IM300). mRNA-injected oocytes were left for 2–5 hours or overnight before fertilization and imaging of fusion protein constructs. Epifluorescence imaging was performed as previously described [28]. Briefly, time-lapse imaging of Venus, Rfp1 and mCherry constructs was performed on an Olympus IX70 inverted microscope set up for epifluorescence imaging. Sequential brightfield and fluorescence images were captured using a cooled CCD camera (Micromax, Sony Interline chip, Princeton Instruments, Trenton NJ) and data collected was analysed using MetaMorph software (Molecular Devices, Sunnyvale CA). Confocal imaging was performed on a Leica SP2 or Leica SP5 confocal microscope at 19°C.

### MBP Kinase and Histone H1 Kinase Measurements

The Myelin Basic Protein kinase activity and histone H1 kinase activity were assayed as described previously [30]. Samples of 5 oocytes were collected by first washing the oocytes through 1 M glycine (pH 8) three times to remove the sea water (this does not alter meiotic progression). The oocytes were then removed in a volume of 1 µl and transferred to 4 µl reaction buffer (25 mM Hepes, 80 mM b-glycerophosphate, 5 mM EGTA, 10 mM MgCl_2_, 1 mM DTT, 10 mg/ml leupeptin/pepstatin/aprotinin, 0.2 mM AEBSF, 1 mM benzamidine, 100 mM NaVO_4_, 5 mM NaF, pH 7.2). At this point, the oocytes were snap-frozen in liquid nitrogen. After defrosting, samples were treated exactly as described previously [21].

## Results

### Characterization of Ascidian Aurora, TPX2 and INCENP

**Figure 1:**
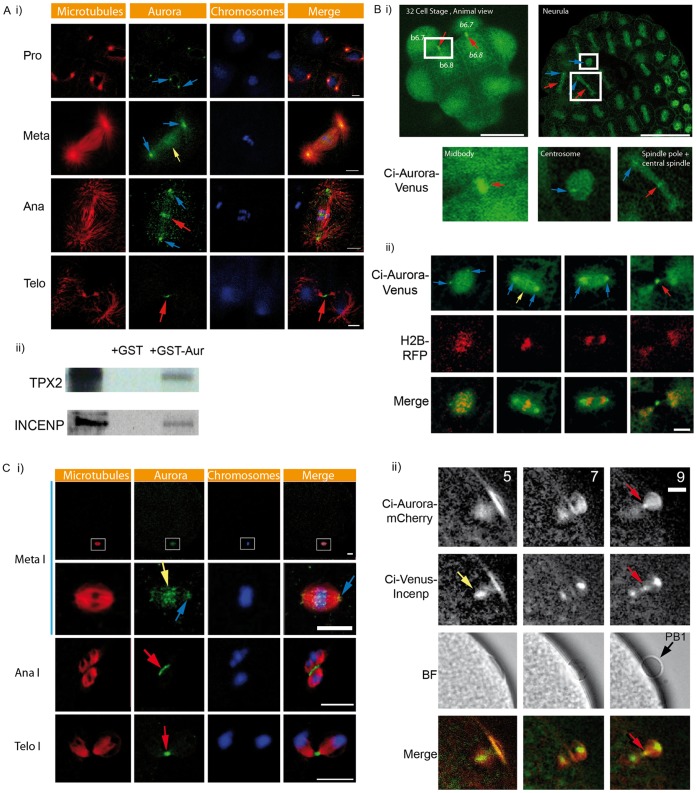
Characterization of Aurora localization during cleavage cycles and polar body extrusion. **A**) i) Confocal images of the endogenous localization pattern of Aurora in *P. mammillata* embryos during one mitotic cycle. Aurora is localized in prophase to both centrosomes (blue arrows). At metaphase Aurora is strongly localized to both spindle poles (blue arrows) and weakly to the metaphase chromosomes (yellow arrow). At anaphase Aurora localizes to the spindle poles (blue arrows) and the spindle midzone (red arrow). At telophase Aurora localizes to the midbody (red arrows). Aurora was labeled with an anti-Aurora antibody (green), DAPI staining shows DNA/chromosomes (blue) and microtubules labeled with anti-tubulin antibody are depicted as red. Scale bars  = 10 µm. ii) GST-Aurora pull downs showing interaction between Ci-Aurora and Ci-TPX2 and Ci-INCENP. n = 3 experiments. **B**) Confocal images of live cleavage stage embryos showing Ci-Aurora::Venus localization pattern. i) A surface view of a 32 cell stage embryo shows that Aurora localizes strongly to the midbody (red arrows). Sister blastomeres (b6.7 and b6.8) are indicated on the right and left sides of this embryo (these embryos are bilaterally symmetrical). The boxed region has been enlarged to show the midbody labeling in greater detail. Confocal image of a neurula stage embryo showing Ci-Aurora::Venus localization to the centrosome (blue arrows), spindles (appear green), spindle poles (blue arrows) and the central spindle (red arrows). The boxed areas have been enlarged to highlight the centrosome, spindle and central spindle localization. Scale bar  = 50 µm. ii) Confocal images of Ci-Aurora::Venus (green) and histone H2B::Rfp1 (red) localization during one mitotic cycle in a cleavage stage embryo. Ci-Aurora::Venus localizes to both centrosomes (blue arrows), spindle poles (blue arrows), weakly to the chromosomes (yellow arrow), and the midbody (red arrow). Merged images are shown. Scale bar  = 10 µm. **C**) i) Confocal immunofluorescence images of endogenous Aurora (green), microtubules (red) and DNA (blue) stained with DAPI during first polar body extrusion. Aurora is enriched at the spindles poles (blue arrows), the chromosomes (yellow arrow), the spindle midzone (red arrow) and the midbody (red arrow). n  = 43 oocytes, 3 animals. Scale bars  = 10 µm. ii) Epifluorescence images showing Ci-Aurora::mCherry and Ci-Venus::INCENP localization through metaphase I to first polar body extrusion. Ci-Aurora::mCherry is localized on the metaphase I spindle (5 min.) then the midbody (9 min., red arrow). Ci-Venus::INCENP localization on metaphase I chromsomes (5 min., yellow arrow) then the midbody (9 min., red arrow). Corresponding bright field images showing polar body extrusion at 7 and 9 min. Merged fluorescence images showing midbody co-localization of Ci-Aurora::mCherry (red) and Ci-Venus::INCENP (red arrow). n  = 9 oocytes, 3 animals.

One isoform of Aurora is present in the ascidians rather than the two isoforms present in the vertebrates. In order to investigate the localization of the unique isoform of Aurora in ascidians, we studied the localization of endogenous Aurora and compared it with the localization pattern observed with Aurora fluorescent fusion protein constructs (Venus and mCherry) during meiotic and mitotic cell cycles in cleaving embryos. In *P. mammillata* cleavage stage embryos Aurora is enriched at the centrosome, spindle and spindle poles, chromosomes, spindle midzone, and the midbody (Figure 1Ai). An identical localization pattern is found with Aurora fluorescent fusion protein constructs (Figure 1B). Briefly, Ci-Aurora::Venus is enriched at the centrosomes, spindle and spindle poles, chromosomes, spindle midzone, and midbody (Figure 1B). In oocytes endogenous Aurora is also enriched at the chromosomes, central spindle and midbody (Figure 1C, red arrows). Ci-Aurora::Venus also labels the midbody in live oocytes during first polar body extrusion (Figure 1Cii, red arrow). We used the chromosomal passenger protein INCENP as a marker of the midbody (Figure 1Cii). Both Ci-Venus::INCENP and Ci-Aurora::mCherry co-localize at the midbody during first polar body formation approx. 9 min. after fertilization (Figure 1Cii). Finally, we wished to determine whether urochordate ascidian Aurora would interact with ascidian TPX2 and/or INCENP. We found that Ci-Aurora binds to both Ci-TPX2 and Ci-INCENP (Figure 1Aii).

**Figure 2:**
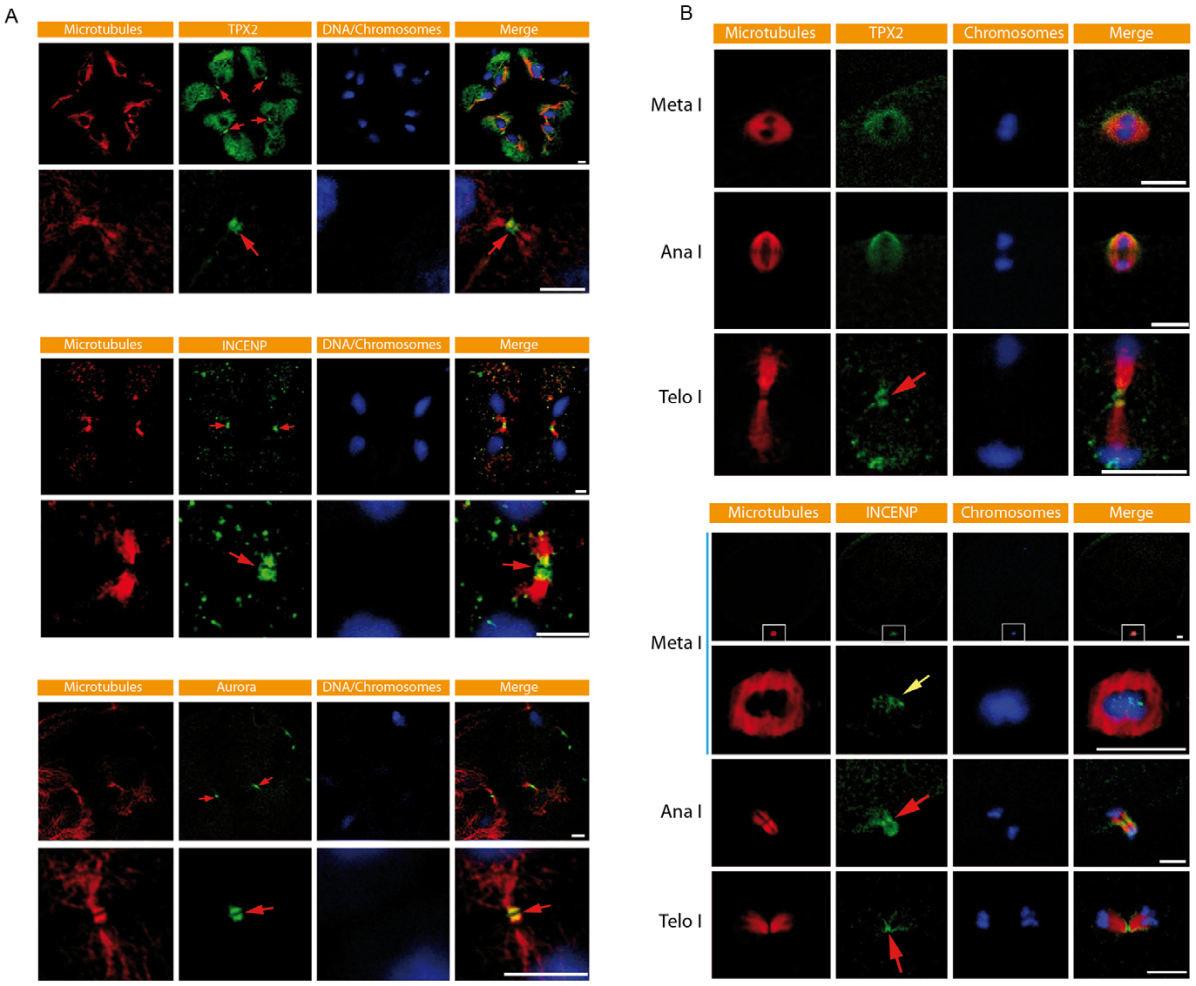
Characterization of the midbody during maternal meiosis and mitosis. **A**) Confocal images of TPX2, INCENP and Aurora immunofluorescence in *Phallusia mammillata* cleavage stage embryos. TPX2, INCENP and Aurora all label the midbody (red arrows) and all display a similar labeling pattern with a central dark zone at the centre of the midbody. Microtubules (red) and DNA/chromosomes (blue) labeled with DAPI are also shown. Scale bars  = 10 µm. **B**) Confocal images of TPX2 and INCENP immunofluorescence during extrusion of the first polar body. TPX2 labels the Meta I spindle and the midbody (red arrow). INCENP labels chromosomes at Meta I (yellow arrow) then the central spindle (red arrow) and midbody at Telophase I (red arrow). For INCENP the meiotic spindle was imaged in cross section in order to reveal INCENP kinetochore labeling of individual bivalent chromosomes more clearly. Scale bars  = 10 µm.

We next examined the localization of TPX2 and INCENP in the ascidian. In cleavage stage embryos TPX2 is clearly present at the midbody (Figure 2, red arrows). Higher magnification shows that TPX2 labeling at the midbody is interrupted by a dark central zone (Figure 2). TPX2-mCherry also localizes to the midbody. Antibodies to INCENP and Aurora also revealed a similar labeling pattern at the midbody (Figure 2, red arrows). In oocytes we also detected TPX2 and INCENP at the midbody during formation of PB1 (Figure 2B). Aurora is also present at the midbody (Figure 1C). Together these data indicate that TPX2 is localized to the midbody during cleavage cycles and during polar body extrusion. To our knowledge this is the first time TPX2 has been found at the midbody in an embryo and during polar body extrusion; TPX2 localization has been examined in detail in *Xenopus* egg extracts and *Xenopus* tissue culture cells and it is not found at the midbody [4].

### Localization of Ascidian Aurora to Spindle and the Midbody via TPX2

**Figure 3:**
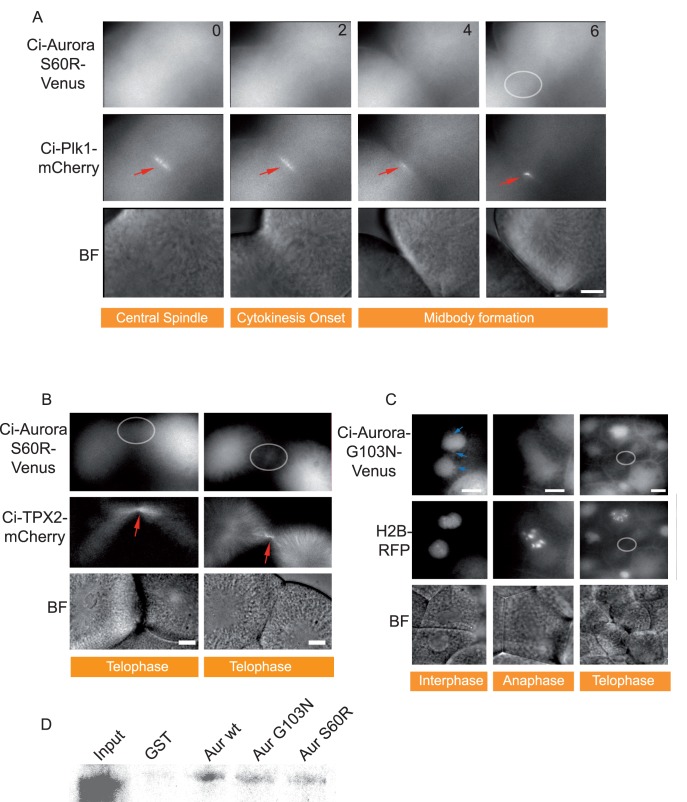
Two different point mutations of Ci-Aurora prevent midbody localization. **A**) Epifluorescence images from a 4D time-lapse series (time in min. is indicated). Point mutation of Ci-Aurora S60R::Venus prevents localization to the spindle pole as expected. However, midbody localization is also perturbed. Ci-Plk1::mCherry localization to the central spindle and midbody is indicated (red arrows) while Ci-Aurora S60R::Venus shows no midbody localization in the same cell. Corresponding brightfield images are shown. Scale bar = 10 µm. n = 9, 3 animals. **B**) Epifluorescence images from a 4D time-lapse series. Point mutation of Ci-Aurora S60R::Venus prevents midbody localization. Ci-TPX2::mCherry localizes to the midbody (red arrow) while Ci-Aurora S60R::mCherry is completely absent from the midbody at telophase. Corresponding brightfield images are shown. Scale bar  = 10 µm. n = 9, 3 animals. **C**) Epifluorescence images from a time-lapse sequence showing that point mutation of Ci-Aurora G103N::Venus prevents midbody localization (grey circle) during telophase. Note that centrosome localization during interphase is preserved (blue arrows). HH2B::Rfp1 was used to precisely follow the mitotic cycle. Note also that AuroraG103N::Venus did not localize to the chromosomes. Corresponding brightfield images are shown. Scale bar  = 10 µm. n = 23, 3 animals. D) GST-CiAur^wt^, CiAur^G103N^ and CiAur^S60R^ were retained on the Sephadex beads for later interaction with radioactive ^35^S-labeled CiTPX2. More TPX2 was found attached to Aur^wt^ than to either Aur^G103N^ or Aur^S60R^. Three experiments.

Since TPX2 has not been extensively examined in the invertebrates, we explored the possibility that Ci-TPX2 would localize Ci-Aurora to the spindles. We therefore constructed two mutants that had been reported to prevent Aurora interaction with TPX2 in vertebrates and hence that perturbed spindle localization of Aurora-A kinase but not localization to the centrosome (AURKA^G198N^: [31]; AURKA^S155R^: [32]). The equivalent amino acids in Ci-Aurora were mutated (S60R and G103N). Neither Ci-Aurora^S60R^::Venus nor Ci-Aurora^G103N^::Venus localized to the spindles but centrosome localization was retained for AURKA^G198N^ (Figure 3) exactly as predicted from studies in vertebrates [31]. These data strongly suggest that Aurora localization to the spindle but not the centrosome in urochordate ascidians is mediated by TPX2 as in the vertebrates. TPX2-independent centrosome localization has also been reported for starfish Aurora in HeLa cells [6]. Surprisingly, we found that neither Ci-AURK^G103N^ nor AURKA^S60R^ localized to the midbody (Figure 3A,B, C grey circles), which were simultaneously labeled with Ci-Plk1::mCherry (Figure 3A). Mutant forms of Ci-Aurora that perturb localization of Ci-Aurora to the midbody were found to interact less strongly with TPX2 (Figure 3). This is similar to findings in HeLa cells where Aurora-A^G198N^ still binds to TPX2 but with less affinity than Aurora-A^wt^ [31]. These data suggest the interesting possibility that urochordate ascidian Aurora localization to the midbody is mediated, at least in part, by TPX2. It is also interesting to note that we did not observe an increase in localization to the chromosomes of the mutated AURK^G103N^ as we may have expected from work in vertebrates [31]. This is consistent with our observations that TPX2 localizes strongly to the midbody in ascidians (Figure 2A). Neither of these mutated forms of Ci-Aurora inhibited cytokinesis, likely because the endogenous protein is still present, suggesting that these mutants do not act as dominant negatives at the expression levels used in our experiments.

### Role of Aurora at the Midbody During First Polar Body Extrusion

**Figure 4:**
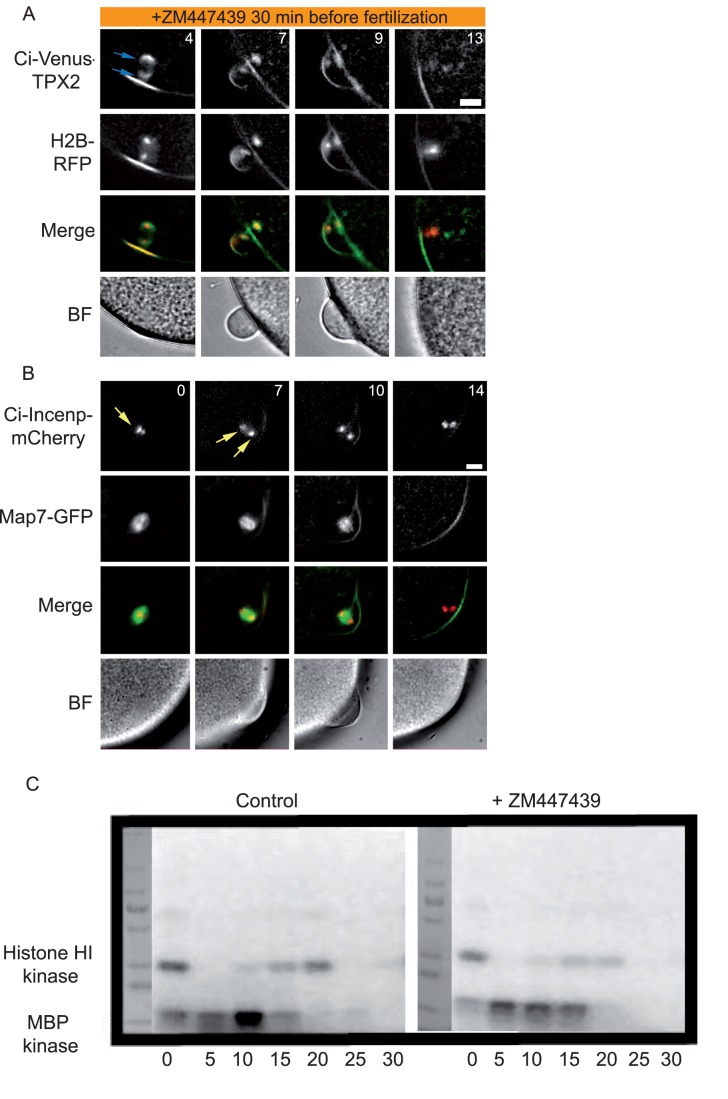
Inhibition of Aurora prevents first polar body extrusion. Unfertilized metaphase I arrested eggs were treated with 10 µM ZM447439 for 30 min. and then fertilized. **A**) Epifluorescence images of Ci-TPX2::Venus to label the spindle poles (blue arrows) and HH2B::Rfp1 to label the chromosomes. Following fertilization chromosomes segregate (4 min., HH2B::Rfp1 image) and the first polar body is extruded (7 min.) but is then re-absorbed (9 min.). Note also a common imaging arfefact in the H2B::Rfp image where there appears to be more H2B::Rfp on the cortex above the meiotic spindle at 4 min. This is likely caused by the curvature of the membrane and can be seen in most fluorescence images at this time. Corresponding brightfield images showing polar body extrusion and re-absorption. n = 17; 3 animals. **B**) Unfertilized metaphase I arrested eggs were treated with 10 µM ZM447439 for 30 min. and then fertilized. Metaphase I chromosomes labeled with Ci-INCENP::mCherry (yellow arrows). Following fertilization anaphase I occurs (7 min.) and the spindle appears positioned normally (7 min.). The first polar body is extruded but is then re-absorbed (10 min.) No Ci-INCENP::mCherry midbody labeling is present at this time. n = 9, 3 animals. All scale bars  = 10 µm. **C**) Histone H1 kinase activity (reflecting MPF activity) and MBP kinase activity (reflecting MAPK kinase activity) in control batches of eggs and in eggs bathed in 10 µM ZM447439. No difference in the activities of either kinase was detected. Three experiments.

We focused on the role Aurora plays at the midbody for two reasons. First, to study the function of vertebrate Aurora kinases (A and B) during late mitosis is hindered by the other roles Aurora kinases plays during earlier steps of mitosis, necessitating the use of fast-acting drugs [19]. We used the Aurora inhibitor ZM447439 which we compared with a kinase dead form of Ci-Aurora ^T193A/T247D/S248D^ that acts as a dominant negative : both inhibited cytokinesis in cleaving embryos producing multinucleated cells (Figure S1). Ci-Aurora ^T193A/T247D/S248D^ inhibited cytokinesis in 14 out of 14 embryos (Figure S1) and ZM447439 inhibited cytokinesis in a similar fashion in 45 out of 45 embryos (Figure S1). Secondly, during polar body formation it is not known what role Aurora kinases (A, B and C) play at the midbody. As far as we are aware, Aurora-A kinase function at the midbody during first polar body extrusion has not been examined even though Aurora-A kinase localizes to the midbody in bovine oocytes [24]. Since unfertilized ascidian eggs are arrested at metaphase I we could bypass the early effects of Aurora inhibition, notably on spindle formation, chromosome capture and alignment, and so study directly the affect of Aurora inhibition on midbody function during first polar body formation. By using a reporter construct that we found labeled the chromosomes and the midbody during meiosis I (Ci-INCENP), as well as Map7::GFP and Ci-Venus::TPX2 which label microtubules and the midbody (TPX2), we measured the effect Aurora inhibition would have on midbody localization of those reporter constructs and in midbody formation. Inhibition of Aurora with 10 µM ZM447439 prevented polar body extrusion at a late step (Figure 4A, B). Eggs previously bathed in the Aurora inhibitor formed the membrane protrusion that precedes midbody formation by a few minutes (Figure 4A, B). Ci-Venus::TPX2 labeling at the meiosis I spindle poles is not affected by Aurora inhibition (Figure 4A). Anaphase chromosome movements occur on time in the presence of the Aurora inhibitor (Figure 4A). However, at 7–10 minutes after egg activation when the midbody would have normally formed the polar body begins to be re-absorbed into the egg (Figure 4A, B). Histone H2B::Rfp1 was used to label simultaneously the chromosomes (Figure 4A,B). Since the first polar body was re-absorbed in eggs treated with the Aurora inhibitor we wondered whether this was an indirect consequence of perturbed MPF inactivation (Figure 4A, B). Our measurements indicate that polar body re-absorption was not a consequence of an indirect effect on MPF inactivation at anaphase I since ZM447439 did not prevent the fall in MPF activity (Figure 4C); MPF activity behaved as in untreated eggs during meiotic exit in eggs treated with ZM447439 (Figure 4C). We simultaneously measured MAPK activity which also appeared normal (Figure 4C). The bright field images in 3A and 4B show the early phases of polar body extrusion followed by its re-absorption.

### Ascidian Aurora Localization in Non-vertebrate Deuterostomes

**Figure 5:**
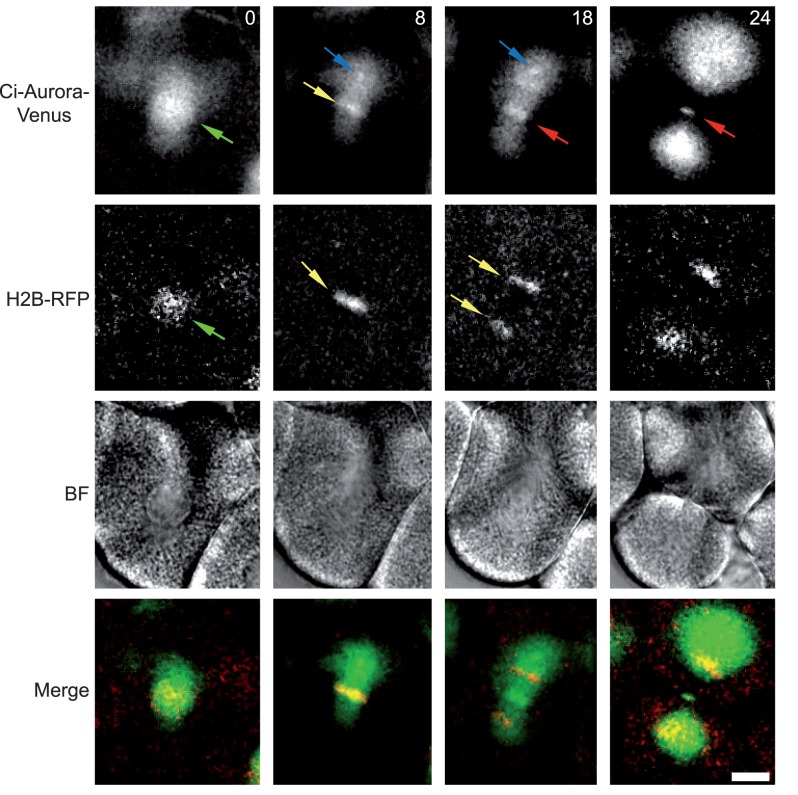
Ci-Aurora::Venus localization in sea urchins. Ci-Aurora::Venus and HH2B::Rfp1 mRNA were injected into unfertilized sea urchin eggs (*Paracentrotus lividus*) and the eggs subsequently fertilized. During cleavage cycles Ci-Aurora::Venus and HH2B::Rfp1 proteins are produced and become fluorescent. Confocal images from a time series of one mitotic cleavage cycle are depicted (at 0, 8, 18, 24 min.). Ci-Aurora::Venus localizes to the nucleus (green arrow), chromosomes (yellow arrow), the spindle pole (blue arrow) and finally to the central spindle and midbody (red arrow). Corresponding brightfield and merged images are shown (red is HH2B::Rfp1 and green Ci-Aurora::Venus). Scale bar is 10 µm. n = 16, 3 animals.

Finally, we were intrigued to determine whether ascidian Aurora would localize in lower deuterostome echinoderms as in the ascidian. Echinoderms also contain one isoform of Aurora kinase [6]. Figure 5 shows the localization pattern of Ci-Aurora in cleavage stage sea urchin *Paracentrotus lividus* embryos together with histone H2B::Rfp1 in order to precisely determine cell cycle phases. Ci-Aurora::Venus localizes to the spindle (Figure 5 blue arrow), chromosomes (Figure 5, yellow arrow), central spindle (Figure 5, red arrow) and the midbody (Figure 5 red arrow).

## Discussion

### Non-vertebrate Deuterostome Aurora and TPX2

**Figure 6:**
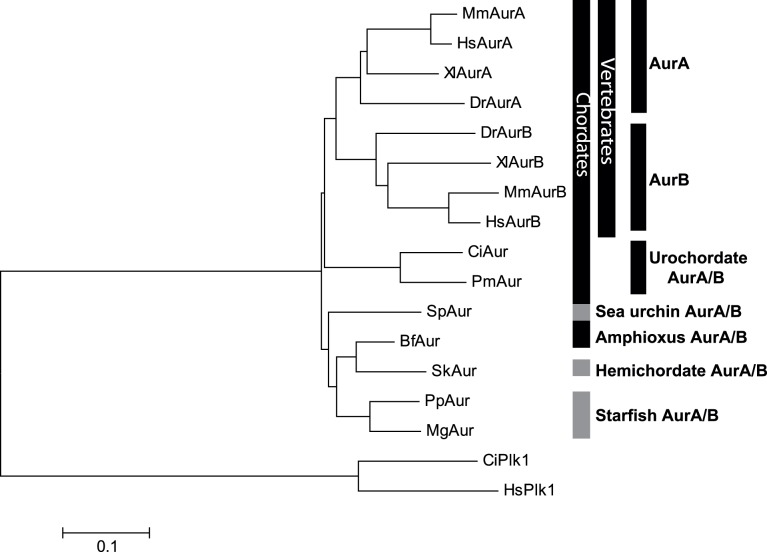
Phylogenetic tree of deuterostome Aurora-A, Aurora-B and Aurora-AB kinases rooted by Plk1 kinases. Phylogenetic tree constructed using CLUSTALW in BioEdit to align the catalytic domains of deuterostome Auroras. The tree was produced using MEGA employing the neighbor joining method. Species represented :HsAur A and B (human), MsAur A and B (mouse), XlAur A and B (*Xenopus laevis*), DrAur A and B (*Danio rerio*), CiAur (*Ciona intestinalis*, ascidian), PmAur (*Phallusia mammillata*, ascidian), SpAur (*Strongylocentrotus purpuratus*, sea urchin), SkAur (*Saccoglossus kowalevskii*, hemichordate), BfAur (*Branchiostoma floridae*, cephalochordate), MgAur (*Marthasterias glacialis*, starfish), and PpAur (*Patiria pectinifera*, starfish). Human and *Ciona* Polo like kinase 1 were used as the outgroup.

All non-vertebrate deuterostomes that we analyzed contained one isoform of Aurora-kinase that contained the Gly residue common to vertebrate Aurora-A that mediates binding to TPX2 (Figure 6). In vertebrates Aurora-A kinase binds to TPX2 [4], [33] which targets Aurora-A kinase to the mitotic spindle but not to the midbody [4], [33], [34]. TPX2 was originally thought to be unique to vertebrates [4], but a *C. elegans* TPX2-like protein (TPXL-1) was identified that was capable of interacting with Aurora-A kinase (AIR1) [35]. TPX2 has since been identified in *Drosophila* [36] and in plants [34]. In vertebrates TPX2 is not found at the midbody and indeed is degraded in HeLa cells before the midbody forms by the APC/C^Cdh1^ (anaphase promoting complex/cyclosome) [37]. However, in *Xenopus* cell free extracts TPX2 is still present during G1 [38] and labels the microtubules but not those microtubules nearest the midbody [4]. It was therefore somewhat intriguing to find TPX2 at the midbody in the ascidian, so we explored its role a little further by creating two separate point mutations of ascidian Aurora predicted to prevent interaction with TPX2 [31], [32]. Both mutated forms of Aurora failed to localize to the spindle but did still localize to the centrosome (Figure 4C) as predicted from results in vertebrate cells indicating that ascidian Aurora behaves much like its vertebrate counterpart [31], [32]. However, neither of the mutated forms of Aurora localized to the midbody in cleavage stage ascidian embryos, suggesting that in the ascidian TPX2 binding is required for Aurora midbody localization. Interestingly, since neither of the mutated forms of Aurora localized to chromosomes we also suggest that we may also have perturbed binding to INCENP (although we failed to verify this in our in vitro pull down assays). This is intriguing since one of the Aurora mutants (G103N) should have increased affinity for INCENP according to one previous study performed in vertebrates [31].

### A Role for Aurora-A Kinase at the Spindle Midzone and Midbody

Identifying all the specific roles played by Aurora-A kinase in somatic cells, embryos and oocytes has been difficult. In somatic cells Aurora-A kinase destruction participates in mitotic exit and cytokinesis. Aurora-A kinase is localized to the spindle midzone in HeLa cells [39]. Careful measurement of the kinetics of Aurora-A kinase and TPX2 levels relative to the time of telophase has shown that both Aurora-A kinase and TPX2 are present during the beginning of telophase in HeLa cells [39]. Aurora-A kinase then TPX2 are destroyed by the APC/C^Cdh1^ [39]. Aurora-A kinase destruction is necessary for correct mitotic exit since over-expression of Aurora-A kinase in HeLa cells causes the formation of broad cytoplasmic connections, cytokinesis failure [40] and spindle elongation [39]. In somatic cells it is therefore thought that Aurora-A kinase must be destroyed for proper mitotic progression.

However, in many oocytes and cleavage-stage embryos the APC/C^Cdh1^ is not active and Aurora-A kinase levels remain elevated during mitotic exit. For example, in mouse oocytes Aurora-A is the most abundant form or the three Aurora-kinases A, B and C [22] and is stable during meiosis I when the first polar body is being extruded [23]. TPX2, the binding partner of Aurora-A, is also stable during meiosis I and II in mouse oocytes due to absence of APC/C^Cdh1^, and its knockdown prevents first polar body extrusion [41]. Moreover, in bovine oocytes Aurora A localizes to the midbody during first polar body extrusion [24]. In mouse oocytes Aurora-A is enriched at the spindle poles and at the spindle midzone during meiosis I [23]. Although these observations warrant investigation, the role of Aurora-A at the spindle midzone has not been analyzed during first polar body extrusion. This is likely because Aurora-A kinase is multifunctional so knockdown of Aurora-A results in a disorganized MTOCs (microtubule organizing centers) and misalignment of chromosomes during meiosis I [22]. Here we find that inhibition of ascidian Aurora causes the first polar body to re-absorb (as well as blocking cytokinesis in embryos). Our data are consistent with the view that ascidian Aurora functions at the midbody to help facilitate contractile ring constriction. In *C. elegans* AIR2 (Aurora-B) contributes to contractile ring constriction through a mechanism parallel to the centralspindlin component MKLP1 [42]. But what role might TPX2 play at the midbody? It is interesting to note that TPX2 was recently found at the midbody in *Xenopus* egg extracts and to act as a scaffold protein for the CPC where it enhances Aurora B activity [43]. However, Aurora-A kinase localization was not documented in this article.

In the light of our findings we suggest that it will be interesting to re-assess the role played by Aurora-A kinase for spindle midzone and midbody function in the vertebrates, particularly in oocytes and early embryos where the APC/C^Cdh1^ is not yet active.

## Supporting Information

Figure S1 **Kinase dead Aurora and ZM447439 prevent cytokinesis.** Confocal images of embryos previously injected with mRNA encoding kinase dead dominant negative Ci-Aurora (T193A/T247D/S248D), termed KD-Aurora. Microtubules are labeled with Map7::GFP (green) and chromosomes with histone H2B::Rfp1 (red). The effect of the KD-Aurora were indistinguishable from Aurora inhibition with 10 µM ZM447439. Both cause the appearance of multiple nuclei in the cells due to the inability of the cells to complete cleavage. n = 14, 3 animals for kinase dead Aurora (14/14 cytokinesis inhibited) and n = 45, 7 animals for ZM447439 (45/45 cytokinesis inhibited). Scale bars  = 10 µm.
(EPS)
